# Toward T Cell-Mediated Control or Elimination of HIV Reservoirs: Lessons From Cancer Immunology

**DOI:** 10.3389/fimmu.2019.02109

**Published:** 2019-09-10

**Authors:** Geetha Mylvaganam, Adrienne G. Yanez, Marcela Maus, Bruce D. Walker

**Affiliations:** ^1^Ragon Institute of MGH, MIT and Harvard, Cambridge, MA, United States; ^2^MGH Cancer Center, Boston, MA, United States; ^3^Howard Hughes Medical Institute, Chevy Chase, MD, United States; ^4^Institute for Medical Engineering and Sciences, MIT, Cambridge, MA, United States

**Keywords:** HIV, cancer, remission, CTL (cytotoxic T lymphocyte), immunothearpy

## Abstract

As the AIDS epidemic unfolded, the appearance of opportunistic infections in at-risk persons provided clues to the underlying problem: a dramatic defect in cell-mediated immunity associated with infection and depletion of CD4^+^ T lymphocytes. Moreover, the emergence of HIV-associated malignancies in these same individuals was a clear indication of the significant role effective cellular immunity plays in combating cancers. As research in the HIV field progressed, advances included the first demonstration of the role of PD-1 in human T cell exhaustion, and the development of gene-modified T cell therapies, including chimeric antigen receptor (CAR) T cells. In the intervening years, the oncology field has capitalized on these advances, effectively mobilizing the cellular immune response to achieve immune-mediated remission or cure of previously intractable cancers. Although similar therapeutic advances have not yet been achieved in the HIV field, spontaneous CD8^+^ T cell mediated remission or functional cure of HIV infection does occur in very small subset of individuals in the absence of anti-retroviral therapy (ART). This has many similarities to the CD8^+^ T cell mediated functional control or elimination of cancers, and indicates that immunotherapy for HIV is a rational goal. In HIV infection, one major barrier to successful immunotherapy is the small, persistent population of infected CD4^+^ T cells, the viral reservoir, which evades pharmacological and immune-mediated clearance, and is largely maintained in secondary lymphoid tissues at sites where CD8^+^ T cells have limited access and/or function. The reservoir-enriched lymphoid microenvironment bears a striking resemblance to the tumor microenvironment of many solid tumors–namely high levels of anti-inflammatory cytokines, expression of co-inhibitory receptors, and physical exclusion of immune effector cells. Here, we review the parallels between CD8^+^ T cell-mediated immune control of HIV and cancer, and how advances in cancer immunotherapy may provide insights to direct the development of effective HIV cure strategies. Specifically, understanding the impact of the tissue microenvironment on T cell function and development of CAR T cells and therapeutic vaccines deserve robust attention on the path toward a CD8^+^ T cell mediated cure of HIV infection.

## Introduction

Human immunodeficiency virus (HIV) remains one of the most pervasive global health challenges of our time. Currently there are an estimated 37 million persons infected with HIV worldwide with more than 35 million AIDS-related deaths to date (1). The development of combination anti-retroviral therapy (ART) has mitigated the severity of this disease, significantly improving survival rates and life expectancy for persons infected with HIV.

Despite these encouraging developments, the number of new HIV infections has remained largely static and co-morbidities including cancers continue to develop in HIV treated individuals. Furthermore, individuals must remain on life-long therapy due to the persistence of latently-infected CD4^+^ T cells, intractable to ART and immune detection due to proviral integration into the host chromosome and being transcriptionally silent, and due to sequestration in anatomical sites largely devoid of HIV specific CD8^+^ T cells [reviewed in (2)]. In particular, secondary lymphoid sites, such as the gut-associated lymphoid tissue (GALT) and lymph nodes (LN), bear the largest fraction of the HIV burden in ART suppressed individuals (3). Unique microenvironments and distinct compartmentalization of immune subsets within these anatomical sites provide an ideal niche for ongoing viral persistence and limited immune pressure. Although T cell exhaustion and immune escape further hinder the impact of adaptive HIV-specific CD8^+^ T cell responses, there are clear examples of persons who spontaneously control HIV for decades without medications (4), indicating that effective HIV immune containment, if not eradication, can be achieved despite these barriers.

As the HIV field has attempted and largely failed thus far to mobilize the immune system to better prevent, treat, and cure infection, the cancer field has experienced dramatic advances through application of immunotherapeutic interventions that either genetically modify and re-direct T cells or liberate endogenous T cell responses to tumor neoantigens. Remarkable examples of immune-mediated disease-free remissions have been achieved for some previously intractable malignancies, such as melanoma (5–7), non-small cell lung cancer (8, 9), and chemotherapy-refractory leukemia and lymphoma (10, 11). Indeed, key barriers to cancer eradication bear multiple similarities to hurdles experienced in immune control of HIV, such as lack of accessible antigens, chronic immune dysfunction, and tissue microenvironments that impede effective clearance of cancerous cells. The dramatic advances in therapeutic interventions to augment effective CD8^+^ T cell immunity in cancer provide important insights for therapeutic interventions in HIV infection. Here, we discuss the role of CD8^+^ T cell mediated immunity in HIV and cancer, and lessons learned from the advances in cancer treatment that may aid in the development of HIV cure strategies.

## Evidence for CTL-mediated Control of HIV and Cancer

Among the most striking data implicating CD8^+^ CTLs in control of AIDS virus infections come from rapid rebound of viremia following CD8^+^ T cell depletion in the non-human primate (NHP) model of SIV infection (12). These data are supported by human data demonstrating rapid emergence of HIV specific CD8^+^ T cells mediating strong selection pressure concomitant with post peak viral decline (13–15) the observed inverse relationship of HIV-specific CTLs with both viral set-point and rate of CD4^+^ T cell loss (16, 17) and the profound viral control exhibited by a select group of elite controllers who, in the absence of ART, maintain potent HIV-specific T cell responses and do not progress immunologically [reviewed in (4)]. These untreated elite controllers represent <1% of HIV-infected persons, some of whom have been infected for more that three decades and maintain prolonged control of plasma viremia (HIV RNA <50 copies/mL of plasma) (18, 19).

The role of CD8^+^ T cells in this remarkable control of HIV is consistently seen in the context of expression of certain “protective” HLA class I alleles such as B^*^27 and B^*^57, and specific amino acids lining the class I peptide binding groove that present viral peptides for CD8^+^ T cell recognition (20, 21). Containment of viremia in elite controllers has been linked to more polyfunctional CD8^+^ T cells than in persons with progressive disease (22), perhaps in part due to maintenance of virus-specific CD4^+^ T cells (23), as well as enhanced recognition of epitope variants (24).

Complementary evidence of CD8^+^ T cell mediated immune control of HIV also derives from studies of the virus itself. Transmission of amino acid “escape” mutations within the 8–10 amino acid epitopes targeted by CTL is associated with worse outcomes due to replication of pre-adapted viruses (25, 26). Other studies have shown impaired viral fitness due to viral mutations associated with CD8^+^ T cell selection pressure (27, 28). More recent studies indicate that persons who spontaneously control HIV without the need for medication do so at least in part by targeting epitopes containing highly networked amino acids that are critical to structure and function of the virus (29, 30). These sites are highly mutationally intolerant, such that immune driven mutations are likely to impair viral fitness and be less resolvable by compensatory mutations at secondary sites. In addition, HIV infection and depletion of CD4^+^ T cells, with preferential infection of HIV-specific CD4^+^ T cells (31), exacerbates immune impairment by providing insufficient help for HIV-specific CD8^+^ T cells. Indeed, immediate treatment of acute infection leads to preservation of CD4^+^ T cell responses and induction of CD8^+^ T cells with greater functionality (32).

Despite a long history of debate as to whether the immune system plays a role in controlling cancers, particularly of non-viral origin, it is now clear CD8^+^ T cell-mediated immunity is also a major host defense against tumors. In 1909, it was first hypothesized that immune surveillance suppressed the outgrowth of cancers (33), but it took decades to identify cancer neoantigens, giving credence to the idea that tumors could be recognized as foreign (34). Early, *in vitro* studies demonstrated that melanoma-specific CD8^+^ T cells could lyse tumor targets (35). Further evidence included the identification of tumor associated antigen (TAA) expressed on tumor cells but not on normal cells, and the observation that a high frequency of TAA-specific CD8^+^ T cells localized within tumors that spontaneously regressed (36). Density of tumor infiltrating CD8^+^ T cells (TILs) has been shown to negatively correlate with progression of colorectal metastasis (37) and oligoclonal expansions of tumor-infiltrating T cells have been associated with tumor regression (38). Furthermore, the development of checkpoint inhibitors that target and effectively block the PD-1 and CTLA-4 axes have convincingly underscored the importance of endogenous CD8^+^ T cells in the recognition and elimination of tumor cells, but most importantly that the cancer-specific immune response can be manipulated for therapeutic benefit. Of note, this checkpoint blockade-mediated liberation of anti-tumor T cell responses is most effective in tumors that have a high mutational burden (39, 40) [i.e., that result in greater presentation of neo-antigens, especially those with mismatch-repair defects (41, 42)], and in those that upregulate the checkpoint ligands such as PD-L1 (43, 44). In addition, engineered autologous T cells transduced to express synthetic, chimeric antigen receptors, or CAR T cells, have demonstrated that T cells can be engineered to recognize surface antigens present on tumor cells and successfully eliminate the cancer, particularly lymphoid malignancies like B-cell leukemia (45), lymphoma (46, 47), and multiple myeloma (48).

## Mechanisms of CD8^+^ T Cell Immune Failure in HIV and Cancer

Immune failure is a hallmark of cancer and persistent viral infections such as lymphocytic choriomeningitis infection (LCMV), simian immunodeficiency virus (SIV) and HIV. Understanding the mechanisms driving immune dysfunction is critical to the rational development of immunotherapies for the treatment of both HIV and cancer. There are three areas that are particularly relevant to both HIV and cancer, namely immune exhaustion, immune escape, and immunoregulatory factors in the lymphoid tissue (HIV) and tumor microenvironment (cancer).

### Immune Exhaustion

One of the major obstacles to immune control of both HIV and cancers is progressive T cell exhaustion in the face of ongoing pathogen burden. The original demonstration of this phenomenon came from the lymphocytic choriomeningitis virus (LCMV) model (49). Armstrong and Clone 13 LCMV variants result in vastly different immunological outcomes, associated with differences in antigen load and persistence (50). Clone 13 has two nucleotides that differ from LCMV Armstrong, resulting in ineffective clearance by CD8^+^ T cells, chronic viremia, and progressive dysfunction of LCMV-specific CD8^+^ T cells. This includes impaired proliferative capacity and decreased polyfunctionality. Gene expression analysis of virus-specific CD8^+^ T cells revealed upregulation of the negative immunoregulatory molecule PD-1 on these cells in the context of Clone 13 infection compared to Armstrong (49), indicative of immune dysfunction with ongoing antigen persistence. Importantly, the immune exhaustion was shown to be reversible through blockade of the interaction of PD-1 with its ligand PD-L1 or PD-L2.

These features of T cell exhaustion are strikingly similar to what is observed in untreated HIV infection and cancer. Chronic viral infection and cancer are both disease states with inadequate antigen clearance. Memory T cell (T_mem_) development is impaired, and effector T cell (T_eff_) become functionally exhausted with elevated and sustained expression of the check-point receptors like PD-1. The first evidence that reversible T cell exhaustion occurs in humans came from studies of HIV infected persons, and like in the LCMV model, blockade of the interaction with PD-L1 or PD-L2 could at least partially reverse cellular dysfunction (51). In cancer, *in vitro* studies showed that tumor-specific T cells in human melanoma metastases share many features of the exhaustion signature that was characterized in LCMV infection (52). Exhaustion was found to be associated with altered epigenetic and transcriptional profiles, a distinct metabolic signature (53–55) and impaired responses to homeostatic cytokines (56). In HIV infection, PD-1 levels are significantly increased on CD8^+^ T cells during chronic HIV infection, directly correlating with plasma viremia and inversely with CD4^+^ T cell counts (51, 57). It was also found that T cells residing within the LN compartment exhibited even greater levels of inhibitory receptors when compared to the peripheral blood (57) demonstrating anatomical differences in parameters of immune exhaustion, posing the question of how distinct microenvironments shape T cell function. Indeed upregulation of these immunoregulatory ligands on tumor cells is an important mechanism of immune dysregulation (57). Beyond inhibitory receptor expression, the transcriptional and epigenetic profiling of virus-specific and tumor-specific CD8 T cells has revealed key similarities and differences between CD8^+^ T cell responses in the two disease settings. Multiple transcriptional regulators have been associated with CD8^+^ T cell exhaustion, including NFAT, Eomes, BLIMP-1, BATF, FOXO1, FOXP3, IRF4, VHL, c-Maf, implicating various metabolic, and signaling pathways as important contributors to various states of CTL exhaustion [(58–62); reviewed in (50)].

Another consideration in loss of T cell effector functions in HIV and cancer is depletion or diminished activity of antigen-specific CD4^+^ T cells [reviewed in (63, 64)]. These cells enhance CTL expansion, activity, migration, tissue invasion, and memory differentiation. HIV preferentially infects HIV-specific CD4^+^ T cells (31), and loss of these cells is associated with a reversible defect in CD8^+^ T cell *in vitro* proliferation (65). CD27 agonism was shown to recapitulate CD4^+^ T cell help by improving induction of effector CD8^+^ T cells, antigen-specific cell killing, and overall survival in a murine cancer vaccine model (63). Loss of CD4^+^ T cells by HIV infection, or diminished antigen-specific CD4^+^ T cell activity by tumor or virus-induced downregulation of MHC class II impairs induction, expansion, and efficacy of CTL responses capable of viral or tumor clearance, and means to rectify this are needed for both HIV and cancer.

### Immune Escape

Effective primary CD8^+^ T cell responses may drive viral or tumoral evolution, particularly in the context of rapidly mutating pathogens, allowing the outgrowth of variants that are no longer recognized by the host CD8^+^ T cell response. This has been termed CD8^+^ T cell escape in the HIV context [reviewed in (66)], and tumor immunoediting in the cancer field [reviewed in (67)], rendering initial CD8^+^ T cell responses ineffective. Following immune escape, induction of effective *de novo* CD8^+^ T cell responses targeting the mutated epitope or a different epitope is necessary to restore antigen-specific immune control.

In HIV infection, where more than 300 viral epitopes and their restricting class I alleles have been defined, immune escape occurs during the initial period of peak viremia [reviewed in (68)]; moreover, transmission of CD8^+^ T cell immune escape variants is already shaping global viral evolution (69). Of particular relevance to any immunotherapeutic approaches is the finding that the majority of immunodominant CTL epitopes in persons with chronic infection may harbor escape mutations (70), such that simple reversal of CTL dysfunction may be insufficient to augment an antiviral effect.

T cell responses against tumor-associated antigens (TAAs) may be rendered ineffective by immune escape in tumors with high mutation burden. The concept of tumor immunoediting encompasses three phases of the interaction between the protective aspects of adaptive immunity against cancers as well as the “tumor sculpting” functions of the immune response: elimination, equilibrium, and escape (71). TAAs arise from non-synonymous somatic mutations (NSSMs) in protein-coding genes, aberrant expression of an embryonic, placental, testes or other tissue-specific differentiation genes, aberrant overexpression of a wild type gene, and viral proteins expressed by cancer cells. In contrast, the high mutability of HIV is due to the infidelity of the viral reverse transcriptase, which induces errors during the process of converting incoming viral RNA into proviral DNA. On the positive side, mutations that escape adaptive immune surveillance may also inflict a fitness cost to the virus or cancer cell and thus serve to the advantage of the host. And in the HIV context, predictable mutations that arise under immune selection pressure can be incorporated into vaccine immunogens, such as is currently being tested in an efficacy trial of a mosaic vaccine (72).

### Tumor and Lymph Node Microenvironments

**Figure 1:**
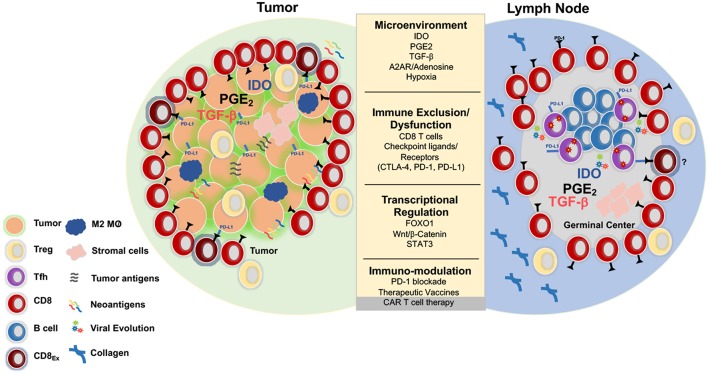
Parallels between immunoregulation in solid tumors and lymph nodes. Tumors and LNs are composed of stromal and immune cells that secrete cytokines and growth factors such as transforming growth factor b (TGFβ), prostaglandin E2 (PGE2), indolamine 2-3-dioxygenase (IDO), and adenosine that shape the tumor and LN microenvironment and collectively contribute to suppression of the T cell response. Adenosine signals through the adenosine 2A receptor (A2AR) and promotes production of cyclic AMP, which impairs T cell trafficking, proliferation, and cytotoxicity (73). Immunosuppression is also induced by regulatory T cells (Treg) that express higher levels of CTLA-4, an inhibitory receptor that outcompetes CD80/CD86 on the surface of effector cells and promotes the production of IDO, an enzyme that degrades tryptophan and leads to impaired proliferation and Treg differentiation. Additionally, Tregs express high levels of CD73/CD39, enzymes that convert ATP to adenosine, which inhibit immune function (74, 75). Tumors upregulate inhibitory ligands such as PD-L1 that bind to inhibitory receptors resulting in suppression of adaptive immune responses (75). Several strategies have been developed in the immune-oncology field to overcome these barriers such as checkpoint blockade, small molecule inhibitors, therapeutic vaccines, and CAR T cell therapy. These immune based therapies can all be extended to the HIV cure field. Several mechanisms that result in resistance to checkpoint blockade and CD8 T cell exclusion include activation of the WNT/β -catenin pathway (76), localization of M2 macrophages within the tumor (77), and the secretion of TGFβ (78).

One of the shared challenges for CD8^+^ T cell mediated clearance of HIV or cancer is the need for migration into and induction of effector function within immunosuppressive tissue environments. In HIV infection, this involves the lymph node microenvironment (LNME), whereas in cancer the tumor microenvironment (TME) is the major site of immune engagement (Figure 1).

Lymph nodes (LN) are not only the inductive site for adaptive immune responses, but are a major site of HIV infection [reviewed in (79, 80)]. They are characterized by interaction of lymphocytes and antigen-bearing dendritic cells (DC) within a fibroblastic reticular network (FRC) (81). Localization of DC subsets, stromal cells, and immune cells within the LN in combination with various cytokines, costimulatory signals, secondary metabolites and the amount and nature of foreign antigen (82–84) impact T cell differentiation by establishing distinct microenvironmental niches (85). Moreover, the LNME is largely immunosuppressive, regulating both naïve and pre-activated T cells through the production of indolamine 2–3 dioxygenase (IDO), Prostaglandin E2 (PGE_2_), adenosine 2A receptor (A2AR) agonists and tumor growth factor β (TGFβ) (78, 86).

As the major site of HIV replication is in CD4^+^ T cells, LNs and the gut associated lymphoid tissue are the initial and persistent targets of infection. Importantly, germinal centers (GC) within LN are important anatomic sites for HIV persistence [reviewed in (87)]. Peripheral blood CD4^+^ T cells constitute ~0.2% of the HIV reservoir, whereas lymphoid resident CD4^+^ T cells represent >50% of the overall HIV burden (3). T follicular helper cells (Tfh, defined as CXCR5^hi^ PD-1^hi^ CD4^+^ T cells) accumulate during chronic HIV/SIV infection, and are highly susceptible to HIV infection (88–91), contributing to both viral production and persistence during chronic untreated and treated HIV infection. Importantly, germinal centers largely exclude HIV-specific CTLs (92).

Progressive dismantling of the FRC networks within lymphoid tissue during HIV infection (93), a consequence of profound CD4^+^ T cell loss, results in increased collagen deposition and significant fibrosis (93–95). These alterations restrict access to IL-7 and limit the life-span of naïve CD4^+^ and CD8^+^ T cells within the LN and the overall generation of T cell immunity within the lymphoid tissue. Excessive accumulation of collagen and other extracellular matrix (ECM) components that occur during HIV/SIV infection has been linked to an early induction of an immunoregulatory response within secondary LT such as increased levels of TGFβ (96–98).

In contrast to the limited understanding of the LNME during chronic HIV/SIV, the TME and its corresponding impact on immune function has been well-characterized. The physical and chemical content within the TME such as the extracellular matrix (ECM), fibroblasts, stromal cells, myeloid cells, and immune cells as well as secreted chemokines and cytokines, collectively impact tumor progression and impair immune function either directly or in *trans* (99). The induction and localization of immune subsets such as regulatory T cells (Tregs), myeloid-derived suppressor cells (MDSCs), tumor infiltrating DCs (TIDCs), and tumor-associated macrophages (TAMs) can hinder effector function and CD8^+^ T cell infiltration and actively contribute to the maintenance of CD8^+^ T cell exhaustion. Tumor cell oncogenic pathways, including oncogenic Wnt/ß-Catenin signaling and gain of function MYC have also been shown to impart immunosuppressive signals within the TME that limit T cell recruitment, activation, and infiltration [reviewed in (100)]. Transcriptional regulation through Signal Transducer and Activator of Transcription 3 (STAT3) within CD8^+^ T cells has also been implicated in limiting CD8^+^ T cell recruitment to (101) and cytotoxic function within (102) tumors. Significant metabolic challenges also occur within the TME which impact T cell function and tumor regression including hypoxia, decreased pH, increased levels of extracellular adenosine, high interstitial fluid pressure, and increased extracellular matrix (ECM) stiffness, akin to what is observed during LN fibrosis in chronic HIV infection. Tumor associated hypoxia commonly occurs during the later stages of cancer, but hypoxia inducible factors (HIFs) can be upregulated due to acidification and glycolytic metabolites within the TME. The concerted effort to understand the TME has led to the development of immune based therapies, currently in clinical trials for the treatment of solid tumors [reviewed in (103)].

Limitations to CD8^+^ T cell trafficking act to impede immune clearance in both HIV infection and solid tumors. Through a variety of mechanisms, CD8^+^ T cells appear to be excluded from both solid tumor masses and LN germinal centers. In both HIV and numerous tumors the relative frequency of tumor infiltrating or GC infiltrating CD8^+^ T cells is inversely correlated with disease outcome in numerous cancers and HIV infection, respectively (104–108). Studies have demonstrated that intra-follicular localization of HIV specific CD8^+^ T cells is correlated with lower plasma viremia (106); however, whether the cytolytic function of these CD8^+^ T cells mediates control remains unknown. Studies from patients with follicular lymphomas (FL, tumors situated in LN) indicate that the presence of functional granzyme B^+^ CTLs at the follicular border within the LN correlated with prolonged progression free-survival (109), whereas higher levels of the inhibitory receptor TIM3 on FL CTLs correlated with shorter relapse-free survival (110).

Understanding the immune suppressive elements of the LNME and TME are likely to lead to additional avenues to immunotherapy. For example, one potential mechanism of immunoregulation shared between the LNME and TME is the pleiotropic cytokine TGFβ. TGFβ has been shown to promote immune exclusion, impair immune function, and limit responsiveness to check-point blockade in metastatic urothelial cancer and other tumors (78, 111). Administration of a TGFβ blocking antibody in combination with anti-PD-L1 has been shown to promote T cell localization within tumors and enhance anti-tumor immunity, leading to increased regression (78, 112). Higher levels of TGFβ have also been observed in the LN during progressive HIV infection. These shared observations suggest that immunoregulation via TGFβ might be playing a similar role in restraining CD8^+^ T cell effector function in the LNME and TME. These data demonstrate the potential for CD8^+^ T cells within LN sites to exhibit cytotoxicity. However, further investigation is required to elucidate the conditions under which CD8^+^ T cell cytotoxicity can occur within the LN, which will have direct implications for the development of HIV cure therapeutics.

## Implications for Immunotherapeutic Interventions

Therapeutic vaccines and immune based therapies aimed at achieving durable remission or cure of HIV have garnered significant interest within the HIV cure field with the identification of the first functionally cured individual known as the “Berlin patient” (113, 114) and hopeful second case reported in London earlier this year (115). Both underwent allogeneic hematopoietic stem cell transplant (HSCT) from HIV-resistant *CCR5*Δ*32* homozygous donors, resulting in reduced expression of the CCR5 co-receptor required for HIV entry. Both patients exhibited virological and immunological features of remission and have been considered cured. However, there is limited feasibility in applying HSCT as a standard of care approach to curing HIV due to toxicity, cost, availability of CCR5Δ32 HSCs and continued susceptibility to infection with CXCR4-utilizing strains (116, 117). The profound outcomes observed in these two cases have nevertheless energized efforts to develop safe and effective HIV cure strategies. Since robust immunological remissions occur in the 1 in 300 HIV infected persons (elite controllers), immune based approaches toward a functional cure are in our view the most rational approach. Given that immunotherapeutic interventions have transformed the cancer field, review of those therapeutic successes is likely to provide critical information for advancing HIV immunotherapy efforts.

### Biological Inhibition of Immuno-Regulatory Pathways

Immune check-point inhibitor (ICI) therapy targeting the CTLA-4 and PD-1 pathways has profoundly altered the management of several cancers, significantly enhancing anti-tumor responses and prolonging progression-free survival. CTLA-4 competes with the co-stimulatory molecule CD28 for binding to CD80/86 on antigen presenting cells, resulting in attenuation of T cell signaling. Ipilimumab, a monoclonal antibody to CTLA-4, blocks this interaction and prevents the inhibitory signal, allowing CTL to kill cancerous or virus infected cells. Pembrolizumab and nivolumab, monoclonal antibodies targeting the PD-1 pathway, engage the PD-1 ligand on target cells, resulting in dephosphorylation of TCR proximal signaling and decreased polyfunctionality, cell cycle progression, survival, and effector function (118, 119). Ipilimumab was the first FDA approved ICI, based on studies in advanced melanoma showing a modest improvement in the overall survival of patients previously treated for metastatic melanoma (120). At present, overall response rate of single ICI therapy is only about 30% in most tumor types for which activity has been shown, such as non-small cell lung cancer (NSCLC), renal cell carcinoma, and metastatic melanoma (7, 121, 122). Biomarkers of ICI responsiveness include an immune inflamed tumor phenotype, described as a gene signature of immune related genes (123), pre-existing anti-tumor CD8^+^ T cells (124), low levels of circulating immunoregulatory cells and cytokines such as IL-10 and TGFβ (125), and a high tumor mutational burden which leads to high levels of tumor associated neoantigens, presumably associated with neoantigen-specific T cells (126, 127).

Efforts to better predict treatment outcomes are advancing effective implementation of ICI therapy for cancer. Parameters including displayed increased localization of CD8^+^ T cells to the tumor core, and increased expression of check-point regulators such as PD-L1 expression on tumor stroma have been shown to correlate with positive disease response to ICI (126, 128). Interestingly, these findings parallel the association of follicular infiltrating CD8^+^ T cells within germinal centers during HIV/SIV infection. Higher frequencies within the GC are associated with reduced plasma viral loads during chronic infection, and these cells retain higher levels of inhibitory receptors (105, 106, 108, 129) and are more responsive to anti-PD-1 therapy (130).

Despite the fact that the initial demonstration of ICI leading to augmentation of CTL function came from studies of HIV, the effective use of ICI in HIV infected individuals is yet to be realized. Indeed, most *in vivo* data assessing ICI for the treatment of AIDS virus infection have been generated in the SIV macaque model. *In vivo* PD-1 blockade of progressive SIV infection resulted in an increase in magnitude and quality of SIV specific CD8^+^ T cells (131, 132), anti-viral B cells (131) and a transient decline in plasma viremia—a clear signal but far short of the best outcomes in cancer. A separate study observed a decrease in hyperimmune activation and microbial translocation in macaques treated with anti-PD-1 (133). Several limited case reports demonstrated that PD-1 blockade promoted increased anti-viral immunity in HIV infected patients and was tolerated (134, 135), but toxicity concerns remain.

PD-1 expression on CD4^+^ T cells has also been explored as a potential cellular biomarker of immune cells enriched in active and latent SIV/HIV (89–91, 136–138). *In vitro* studies have described variable effects of PD-1 blockade on disrupting the latent viral reservoir (139), and substantial reactivation of the latent HIV reservoir with anti-PD-1 alone (140) and in combination with the latency reversal agent (LRA) bryostatin (141). In a macaque study, anti-PD-1 administration during suppressive ART led to transient increase in plasma viremia and a reduction in replication competent virus (142). These data suggest that PD-1 signaling may play a role in maintaining viral latency and blockade may allow for disruption and reactivation of the latent viral reservoir. CTLA-4^+^ PD-1^−^ CD4^+^ T cells have also been implicated as a subset of T cells enriched in viral DNA during suppressive ART (143). A recent open-label study found that ascending doses of anti-CTLA-4 were well-tolerated and showed variable changes in detectable plasma viral RNA (144). Check-point blockade monotherapies have elicited modest immunological responses and reactivation of the viral reservoir, suggesting that combination therapeutic approaches may be required for significant destabilization of the HIV reservoir.

We believe that check-point blockade should be considered cautiously as a treatment modality for HIV, as ICIs carry significant toxicity profiles, setting a higher bar when alternative HIV treatments are available. Following ICI therapy for cancer, immune-related adverse events (irAEs), and increased immune cell infiltration into healthy tissues have caused autoimmune-like toxicities. Severe irAEs are more common with ipilimumab (15–43% of patients) than nivolumab or pembrolizumab. However, 10–23% of patients given anti-PD-1 therapy still develop potentially life-threatening toxicities, that increase with co-administration of anti-CTLA-4 (145). A comprehensive meta-analysis conducted to assess irAE's resulting from ICI found higher risk of all-grade rash and colitis with anti-CTLA-4 treatment (146) and a case study of a patient with widespread uveal melanoma had an exceptional response to ipilimumab and nivolumab but suffered severe immune-related sequelae, with identical T cell clones found in the tissues affected (147). Moreover, a recent report assessing patients treated with a single-agent nivolumab or pembrolizumab for advanced cancer found an overall response rate of 82.5% in patients experiencing irAE (148), highlighting autoimmunity as an emerging biomarker for responsiveness to ICI. Thus, there is an ongoing medical need to not only define biomarkers of ICI resistance, but identify mechanisms underlying cross-reactivity and toxicity as well, in an effort to develop therapies that promote remission while limiting immune toxicities.

### Adoptive T Cell Therapy

Chimeric antigen receptor (CAR) T cell immunotherapy has emerged as an important adoptive T cell therapy for the treatment of cancer with the recent FDA approval of the CD19-targeted CAR T cell “living drug,” tisagenlecleucel (Kymriah) for the treatment of adult and pediatric B cell malignancies (45). CARs are synthetic receptors comprised of a single-chain variable fragment (scFV) of an antibody fused to a transmembrane domain and intra-cellular signaling complex [reviewed in (149)]. CAR T cells can re-direct specificity, functionality, and localization of T cells. Clinical trials have shown dramatic outcomes in patients with relapsed, refractory B cell cancers. A phase II clinical trial utilizing the CD19-targeting CAR for the treatment of B cell acute lymphoblastic leukemia (ALL) observed an 81% complete response (CR) rate at 28 days of follow-up, and a relapse-free survival of 59% with a short median 12-month follow-up. Despite initial high rates of remission, a significant fraction of patients will relapse with CD19^+^ or CD19^−^ tumors due to decreased persistence/function of the CAR T cells, antigen loss, and impairment due to the immunosuppressive tumor microenvironment (150). Increased persistence of circulating CAR T cells correlated with durable responses and improved clinical outcomes, indicating that these therapies can be further improved (151). This is especially true for CAR T cells that contain the 4-1BB costimulatory domain, which allows the CAR T cells to primarily utilize oxidative metabolism vs. glycolysis which CD28 costimulatory CARs rely on, allowing for enhanced persistence (152).

Despite persistence of CAR T cells, relapses can occur due to antigen loss post CAR infusion, which accounts for 40% of relapses (153). Moreover, the immunosuppressive tumor microenvironment significantly contributes to poor clinical outcomes by inducing early dysfunction, decreased expansion of CAR T cells, and limited persistence *in vivo* (154). A new generation of CAR T cells is being constructed to overcome these immune barriers. Alternative strategies include the development of CAR constructs targeting antigens other than CD19, the generation of bi-specific CARs that target more than one antigen, cytokine secreting CARs that produce IL-12 (155) and IL-18 (156), or anti-PD-1 (157). Additionally, CAR T cells may be engineered to express chemokine receptors and cytokines to improve their homing and tumor infiltration, but the efficacy of these approaches has not yet been confirmed in clinical trials. One example of this approach is engineering CAR T cells to express IL-7 and CCL19 (158) to enhance survival and T cell trafficking to secondary lymphoid sites, respectively.

Chimeric antigen receptor (CAR) T cell therapy for HIV actually predates its use in cancer, with the first studies completed in the mid 1990's, when a CD4-based CAR, shown to be effective *in vitro* and safe and well-tolerated *in vivo*, provided no clear clinical benefit and no reduction in the peripheral viral reservoir (159, 160). Follow-up studies attributed lack of efficacy to limited CAR T cell persistence, likely due to the high IL-2 dose used in manufacturing. The CAR contained CD4 extracellular and transmembrane domain, which might have increased CAR T cell susceptibility to infection, but lacked costimulatory domains, which could limit cellular functionality (161). Inclusion of costimulatory domains has been shown to be critical for CAR T cell efficacy in cancer. Despite limited function, there were no associated malignancies found with the transduced infused HIV CD4 CARs, which was promising for virally transduced adoptive T cell therapy. In the last several years, a growing number of high affinity broadly neutralizing antibodies (bNAbs) has been identified against HIV passive antibody infusion trials assessing the efficacy of HIV bNAbs have produced modestly decreased viral loads in viremic patients (162), increased clearance of infected cells (163), a delay in viral rebound (164, 165) and viral suppression post treatment interruption in 30% of patients until bNAb titers waned. Moreover, VRC01 and PGT121, bNAbs targeting the CD4 binding site and the V3 glycan of *env*, respectively, blocked HIV-1 replication from reactivated latently infected cells *in vitro* (166).

The growing repertoire of HIV bNAbs and enhanced function and persistence of second and third generation CAR T cell vectors have propelled efforts to design bNAb CARs for HIV remission or cure. Several groups have reported the development of bNAb and CD4 expressing CAR T cells (CD4 CAR) that can effectively limit HIV replication *in vitro* (159, 167–169). Moreover, the recent identification of follicular CXCR5^+^ CD8^+^ T cells and their potential contribution to control of viral replication within GC “hotspots” of active and latent HIV, has led to the development of HIV CAR T cells that over-express the chemokine receptor CXCR5, to promote trafficking into B cell follicles. A proof-of-concept study in macaques showed that infusion of CD8^+^ T cells overexpressing rhesus CXCR5 increased localization within the GC (170). A separate group developed CXCR5+ CAR T cells expressing the CD4 co-receptor for HIV *env* specificity and found the cells functionally capable of limiting SIV infection *in vitro* and chemotaxing in response to CXCL13 in transwell and LN organoid cultures (171). These studies highlight the potential feasibility of developing virus-specific CAR T cells with an increased ability to traffic to specific anatomical sites such as GC that harbor a large fraction of the HIV reservoir.

Several considerations should be taken in developing adoptive cell therapies that enhance CD8^+^ T cell trafficking to and detection of infected CD4^+^ T cells in GC of lymphoid tissue. In contrast to tumor masses, GC/B cell follicles are critical anatomical sites for the induction of systemic immunity. Tfh cells localized within GC not only harbor a significant fraction of the HIV reservoir but are key mediators in the development of humoral immunity. Enhanced infiltration of CD8^+^ T cells into the GC of LN may impact antibody development and pathogen specific immunity. Of note, recent trials assessing the efficacy of re-programmed autologous CAR T cells for the treatment of follicular lymphoma (FL) demonstrated successful restoration of immune function in patients with relapsed or refractory disease (172); however cytokine release syndrome (CRS) and neurotoxicities were experienced in line with what has been observed in other CAR T cell trials. Additional studies to assess the efficacy of ICI in the context of FL found varying objective response rates with some measured as high as 67% (172). However, with limited power in these studies, it is unknown whether ICI treatment of FL comes with a similar toxicity profile to what has been observed in other ICI responsive cancers. These findings suggest that enhanced accumulation of CD8+ T cells within lymphoid sites may be mechanistically supported and immunologically tolerated, but further studies need to be conducted to understand CD8^+^ T cell targeting of LNME and the associated toxicities of targeting cells at LN sites.

Additionally, although the LNME has not been fully characterized in the context of HIV infection, collective data suggest an immunosuppressive environment may hamper the local functionality of CAR T cells. Immuno-oncology strategies to enhance intra-tumoral CAR T cell efficacy may serve to overcome similar constraints of the LNME. Strategies include combining CAR T cells with ICI therapy and development of CAR T cells with endogenous PD-1 knockout or encoding secretable check-point inhibitors and effector cytokines such as IL-18 (156, 173), IL-12 (174), or a tethered IL-15 (175), as well as engineering T cells to express a domain-negative form of the TGFβ receptor (176, 177). A recent *in vivo* trial in macaques using the human IL-15 superagonist ALT-803 demonstrated enhanced trafficking of SIV specific CD8^+^ T cells to B cell follicles. HIV specific CARs with the ability to secrete IL-15 *in situ* may direct localization of these cells within B cell “sanctuary sites.” Additionally, there is potential for viral escape following treatment with HIV-specific CAR T cells that target a single HIV *env* epitope, similar to what was observed in phase 1 studies assessing single infusions of bNAbs targeting distinct HIV-1 envelope epitopes (164, 178). However, more recent studies have found that despite selection of escape variants, rebound viruses did not show further resistance to other antibodies that targeted different envelope epitopes (164, 165, 178) and combination approaches with multiple bNAb scFVs can maintain viral suppression (179) and limit viral resistant variants. Thus, combination multi-specific CAR T cell approaches could be taken to promote durable viral control. Further studies need to be conducted to assess the *in vivo* potential of the single and multi-specific bNAb CAR T cells to reduce the HIV reservoir and mediate post-treatment viral control. What is very clear is that advances in CAR T cell therapy for cancer and HIV will benefit both.

### Therapeutic Vaccines

The rationale for therapeutic vaccines is similar for cancer and for HIV: for both tumors and viruses, genomic heterogeneity limits the efficacy of naturally induced immune responses, and in both diseases, there is compelling evidence revealed by next generation sequencing and advances in bioinformatics to suggest that targeting the CD8^+^ T cell response to specific epitopes may be beneficial. Moreover, in both diseases there is evidence for dysfunctional natural immune responses that might be countered by therapeutic immunization. For cancer, there are now multiple vaccines that have been licensed by the FDA, and exciting new advances from early phase human clinical trials targeting cancer neoantigens (180), but for HIV, despite some promising results in monkeys infected with SIV, human studies have mostly been disappointing. This is thus an overlap area that deserves considerable attention.

The first attempt at therapeutic vaccination for cancer came over a century ago with the administration of bacterial toxin directly into tumors, which led to tumor regression in a person with an advanced sarcoma (181). This was the first evidence that a tumor-specific immune response could be augmented by immunization. In the cancer field today, there are multiple licensed therapeutic vaccines, starting in 2010 with FDA approval of Sipuleucel-T (Provenge; Dendreon), an autologous dendritic cell vaccine for prostate cancer (182). Autologous and allogeneic tumor vaccines have been tested in different cancer modalities, enhancing anti-tumor responses and prolonging survival (183, 184). GVAX, the most extensively studied whole cell vaccine which is comprised of irradiated, allogeneic, or autologous pancreatic tumor cells genetically engineered to secrete granulocyte colony stimulating factor (GM-CSF), has been used in pre-clinical and clinical studies in an attempt to stimulate dendritic cell activation and T cell priming (185). Despite an observed increase in anti-tumor immunity (186), a phase II trial with GVAX in combination with cyclophosphamide for the treatment of pancreatic cancer failed to show an increase in overall survival (187). More recent advances have been associated with individualized vaccines using tumor whole exome sequencing to identify autologous neoantigens, which have been shown to be immunogenic for intratumoral CD4^+^ and CD8^+^ T cell responses in early phase clinical trials in glioblastoma (180). Thus, far the impact on disease course has been modest, and none of these approaches has effected a cure or sustained remission.

In the setting of HIV infection, there have been multiple attempts at therapeutic vaccination to augment HIV-specific CD8^+^ T cell responses, but thus far there have been no clear successes in humans. Studies of DC based immunotherapy clinical trials conducted for the treatment of HIV have shown modest immunogenicity and modest impact on viral load (188–191), which has often been difficult to interpret due to lack of appropriate controls. More promising results come from recent studies in NHPs suggest that immune modulation may be possible. Several studies conducted in rhesus macaque models have underscored the importance of generating a robust anti-viral CTL response for therapeutic SIV/HIV control. An epidermally administered DNA vaccine expressing highly conserved elements (CE) of the SIV capsid protein p27 in SHIV infected macaques experiencing chronic but controlled SHIV infection. Macaques experiencing a stronger induction of CE specific responses exhibited lower plasma viral loads (192). In a separate study, Ad26/MVA (recombinant adenovirus 26 serotype (Ad26) prime/modified vaccinia Ankara (MVA) boost) with a TLR-7 (Toll-like receptor 7) adjuvant demonstrated a delay in viral rebound and a 2-log reduction in plasma viral loads post treatment interruption (193), where the breadth of the immune response directly correlated with time to rebound and inversely with plasma viral loads. However, a recent randomized controlled trial utilizing a therapeutic vaccine regimen in HIV infected patients who began cART early during the course of infection, showed a limited induction of anti-viral CD8^+^ T cells, no significant effects on the kinetics of viral rebound, and no reduction in the viral reservoir post discontinuation of ART (194). This was despite the addition of human interleukin (IL)-12p35 and p40 proteins via *in vivo* electroporation to maximize immunogenicity, previously shown in non-human primates to enhance the potency of the HIV DNA based vaccine (195, 196). Moreover, the subjects enrolled in this study were within the acute phase of infection with early cART treatment and potentially better preserved immune function, but still failed to show any effect on post-treatment control.

For both HIV and cancer, epitope mutation resulting in immune escape appears to play a major role in the lack of efficacy of host T cell responses, but recent studies suggest that this property may be exploited to therapeutic benefit. In cancers, neoantigens which can be bioinformatically identified have been used as immunogens and shown promise in early phase human trials (180, 197). In HIV infection, application of network theory to HIV structure has revealed that mutation of epitopes at important network positions disproportionately impairs viral replication capacity and that CD8^+^ T cell targeting of highly networked epitopes distinguishes persons who naturally control HIV, even in the absence of protective HLA alleles (30). These data suggest that targeting mutationally constrained epitopes is a promising approach for vaccine design. Support for induction of immune responses to neoantigen epitopes or highly networked epitopes comes from recent studies showing that a synthetic DNA, multi-neoantigen cancer vaccine in a mouse model drives robust MHC class I CD8^+^ T-cell responses which are able to impact tumor growth (198). It is still unknown whether therapeutic vaccinations alone can increase the magnitude of the HIV specific response to a level that can both detect very low antigen levels in ART treated patients as well as induce durable viral suppression upon ART cessation, but future studies incorporating check-point blockade, cell based therapies, and tissue specific/LNME agonists in combination with therapeutic vaccines to develop a functional, highly potent CTL response may be key to containing or eradicating the latent HIV reservoir.

## Conclusions

HIV remains a significant global health burden and despite the profound efficacy of ART in preventing viral transmission (199), the number of individuals living with HIV and on treatment continues to rise each year and non-AIDS related morbidities are increasing with the duration of HIV and time on ART (200, 201). Given the limited impact of ART on viral “sanctuaries,” there is a critical need to identify immune mechanisms within tissue sites that harbor the HIV reservoir and hinder anti-viral immunity, similar to the need for immune based therapies in cancer to access malignant cells in tissue sites and overcome tumor immunosuppressive environments. In the same way that advances in cancer immunotherapy have resulted in durable remission in patients with seemingly incurable malignancies, there is strong rationale for immune control if not eradication of HIV, given that some persons are able to achieve a state of immune-mediated functional cure of HIV infection without the need for ART. A deeper understanding of common mechanisms of immune dysfunction and exclusion as well as mechanisms of tumor response leading to durable remission, will be critical to attaining a functional state of viral remission or cure in HIV infected patients. These include enhancing T cell trafficking into tumors and lymph node HIV sanctuaries, overcoming immune exhaustion, and escape, reversing tumor and lymph node immunosuppressive environments, and eliciting robust CTL responses against neo-epitopes and highly networked epitopes. Caution, however, must be taken when exploring immunotherapeutic interventions to avoid emergence of autoimmunity and other adverse events. A greater understanding of the immune mechanisms regulating the LN microenvironment and the impact of check-point blockade on CTL function, localization, and viral clearance within the LNME will be crucial to the development of HIV cure strategies. Thus, as the field of cancer immunotherapy progresses, the HIV cure field must take heed in determining what therapeutic interventions will prove safe, effective, and clinically justifiable to explore in HIV infected individuals currently durably suppressed with ART.

## Author Contributions

GM and AY carried out the primary research and equally wrote the manuscript. MM edited the manuscript. BW provided oversight in preparation and editing.

### Conflict of Interest Statement

The authors declare that the research was conducted in the absence of any commercial or financial relationships that could be construed as a potential conflict of interest.
